# Optogenetic Activation of Non-Nociceptive Aβ Fibers Induces Neuropathic Pain-Like Sensory and Emotional Behaviors after Nerve Injury in Rats

**DOI:** 10.1523/ENEURO.0450-17.2018

**Published:** 2018-02-15

**Authors:** Ryoichi Tashima, Keisuke Koga, Misuzu Sekine, Kensho Kanehisa, Yuta Kohro, Keiko Tominaga, Katsuyuki Matsushita, Hidetoshi Tozaki-Saitoh, Yugo Fukazawa, Kazuhide Inoue, Hiromu Yawo, Hidemasa Furue, Makoto Tsuda

**Affiliations:** 1 Department of Life Innovation, Graduate School of Pharmaceutical Sciences, Kyushu University, Fukuoka 812-8582, Japan; 2 Department of Molecular and System Pharmacology, Graduate School of Pharmaceutical Sciences, Kyushu University, Fukuoka 812-8582, Japan; 3 Department of Brain Structure and Function, Research Center for Child Mental Development, Faculty of Medical Sciences, University of Fukui, Fukui 910-1193, Japan; 4 Department of Developmental Biology and Neuroscience, Tohoku University Graduate School of Life Sciences, Sendai, Miyagi 980-8577, Japan; 5 Department of Neurophysiology, Hyogo College of Medicine, Nishinomiya, Hyogo 663-8501, Japan

**Keywords:** Aβ fibers, aversion, neuropathic pain, optogenetics, primary afferents, spinal cord

## Abstract

Neuropathic pain is caused by peripheral nerve injury (PNI). One hallmark symptom is allodynia (pain caused by normally innocuous stimuli), but its mechanistic underpinning remains elusive. Notably, whether selective stimulation of non-nociceptive primary afferent Aβ fibers indeed evokes neuropathic pain-like sensory and emotional behaviors after PNI is unknown, because of the lack of tools to manipulate Aβ fiber function in awake, freely moving animals. In this study, we used a transgenic rat line that enables stimulation of non-nociceptive Aβ fibers by a light-activated channel (channelrhodopsin-2; ChR2). We found that illuminating light to the plantar skin of these rats with PNI elicited pain-like withdrawal behaviors that were resistant to morphine. Light illumination to the skin of PNI rats increased the number of spinal dorsal horn (SDH) Lamina I neurons positive to activity markers (c-Fos and phosphorylated extracellular signal-regulated protein kinase; pERK). Whole-cell recording revealed that optogenetic Aβ fiber stimulation after PNI caused excitation of Lamina I neurons, which were normally silent by this stimulation. Moreover, illuminating the hindpaw of PNI rats resulted in activation of central amygdaloid neurons and produced an aversion to illumination. Thus, these findings provide the first evidence that optogenetic activation of primary afferent Aβ fibers in PNI rats produces excitation of Lamina I neurons and neuropathic pain-like behaviors that were resistant to morphine treatment. This approach may provide a new path for investigating circuits and behaviors of Aβ fiber-mediated neuropathic allodynia with sensory and emotional aspects after PNI and for discovering novel drugs to treat neuropathic pain.

## Significance Statement

Neuropathic pain is a debilitating chronic pain condition occurring after nerve damage. A cardinal, intractable symptom is allodynia, pain caused by innocuous stimuli. However, its mechanistic underpinning remains elusive, and, notably, whether non-nociceptive primary afferent Aβ fibers after nerve injury indeed evokes neuropathic pain-like sensory and emotional behaviors is unknown. Using optogenetics, we show for the first time that optical activation of non-nociceptive Aβ fibers of nerve-injured rats is sufficient to produce morphine-resistant pain-like withdrawal behaviors, excitation of spinal Lamina I neurons, and emotional aversion. This study provides a novel approach to facilitate our understanding of the mechanisms underlying Aβ fiber-mediated neuropathic allodynia with sensory and emotional aspects and to discover new drugs for treating neuropathic pain.

## Introduction

Somatosensory information from the periphery is conveyed to the spinal dorsal horn (SDH) via primary afferent sensory neurons. Primary afferents are broadly divided into two classes: nociceptive and non-nociceptive, which respond to noxious and innocuous stimuli, respectively (Todd, 2010; Braz et al., 2014). The nociceptor classification refers to mainly thin, myelinated Aδ fibers and unmyelinated C fibers, which transmit to the superficial SDH (Laminae I and II). The latter low-threshold mechanoreceptors, such as large-diameter, thick, myelinated Aβ fibers, detect innocuous mechanical stimuli and transmit to the deeper lamina of the SDH. Under normal healthy conditions, innocuous mechanical stimulus, such as light touch, does not produce pain. However, after damage to the nervous system from cancer, diabetes, chemotherapy, or traumatic injury, even normal innocuous mechanical stimuli can produce pain (Colloca et al., 2017). This type of abnormal pain is known as mechanical allodynia and is a cardinal symptom of neuropathic pain, which is the debilitating chronic pain condition that follows nerve damage. Neuropathic allodynia is refractory to the currently available treatments, including opioids (Colloca et al., 2017). Thus, the elucidation of mechanisms for neuropathic allodynia and the development of new therapeutic agents are major challenges.

The mechanisms underlying mechanical allodynia have been investigated in rodent models of neuropathic pain. Previous studies have proposed the role of non-nociceptive Aβ fibers, but it remains obscure (Sandkühler, 2009; Lolignier et al., 2015). This might be due to the lack of research tools to selectively manipulate Aβ fibers in awake, freely moving animals. A study showed a reversal of mechanical hypersensitivity after peripheral nerve injury (PNI) by silencing myelinated A fibers in mice (Xu et al., 2015). However, it is unknown whether non-nociceptive Aβ fiber stimulation indeed produces allodynia-like behaviors in models of neuropathic pain. Currently, von Frey filaments are frequently used for assessing mechanical allodynia and for investigating its mechanisms. However, von Frey testing has disadvantages for investigating allodynia, such as the activation of not only low-threshold mechanoreceptors but also nociceptors (Le Bars et al., 2001). In fact, silencing or ablating nociceptive C or Aδ fibers suppresses the decreased hindpaw withdrawal threshold by the filaments after PNI (Tarpley et al., 2004; Shen et al., 2012; Boada et al., 2014; Iyer et al., 2014; Daou et al., 2016), although other studies have not reported these changes (Abrahamsen et al., 2008; Mishra et al., 2011). Thus, for elucidating the mechanistic underpinnings of neuropathic mechanical allodynia, a new approach that enables selective stimulation of non-nociceptive Aβ fibers in awake, freely moving animals is necessary.

To establish this, we employed an optogenetic strategy. In basic pain research, several studies have reported selective activation or silencing of nociceptive C and Aδ fibers (Carr and Zachariou, 2014; Ji and Wang, 2016; Montgomery et al., 2016), but little is known about non-nociceptive A fibers. In this study, we used a transgenic rat line, W-TChR2V4, in which, within the dorsal root ganglia (DRG), the blue light-sensitive cation channels channelrhodopsin-2 (ChR2) are selectively expressed in innocuous mechanoreceptive A fibers, including Aβ fibers (Ji et al., 2012). In W-TChR2V4 rats, the ChR2-expressing nerve endings in the skin are myelinated and associated with tactile end organs such as Merkel discs and Meissner’s corpuscles (Ji et al., 2012). Using optogenetics in combination with behavioral pharmacology, immunohistochemistry, and electrophysiology, we provide the first evidence that optogenetic activation of Aβ fibers of W-TChR2V4 rats with PNI evokes neuropathic pain-like withdrawal behaviors that were resistant to morphine, induces excitation of Lamina I neurons in the SDH, and produces activation of central amygdaloid neurons and aversion to light illumination. This novel approach could be used to investigate neuronal circuits and behaviors responsible for Aβ fiber-mediated neuropathic allodynia with sensory and emotional aspects, and in the development of novel drugs to treat neuropathic pain.

## Materials and Methods

### Animals

W-Tg(Thy1-COP4/YFP*)4Jfhy (W-TChR2V4: NBRPRat no. 0685) rats (Ji et al., 2012) were supplied by the National BioResource Project-Rat, Kyoto University (Kyoto, Japan). All male W-TChR2V4 rats were aged six to seven weeks (for electrophysiological experiments) and 10–12 weeks (for other experiments) at the start of each experiment and were housed in individual cage at a temperature of 22 ± 1°C with a 12/12 h light/dark cycle (lights on 8 A.M. to 8 P.M.), and fed food and water *ad libitum*. All animal experiments were conducted according to the national and international guidelines contained in the Act on Welfare and Management of Animals (Ministry of Environment of Japan) and Regulation of Laboratory Animals (Kyushu University) and under the protocols approved by the Institutional Animal Care and Use committee review panels at Kyushu University.

### Neuropathic pain models

We used the spinal PNI model with some modifications (Kim and Chung, 1992; Tsuda et al., 2011). In brief, under isoflurane (2%) anesthesia, the left fifth lumbar (L5) spinal nerve was tightly ligated with 5-0 silk and cut just distal to the ligature. The wound and the surrounding skin were sutured with 3-0 silk.

### Light illumination of the hindpaw

Rats were placed on a transparent acrylic plate and habituated for 30–60 min to allow acclimatization to the new environment. The plantar surface of the hindpaw (touching the acrylic plate floor) was illuminated with a blue light emitting diode (COME2-LB473/532/100, Lucir: wavelength, 470 nm; duration, 500 ms; interval, 10 s; 10 times per each ipsilateral and contralateral hindpaw) over the acrylic plate. The light power intensity (12 mV) was measured by a thermopile (COME2-LPM-NOVA, Lucir), which was a laser power meter with 12 mW at the skin. Withdrawal responses of the hindpaw to light illumination (0: no reaction, 1: mild movement without any lifting and flinching behaviors, 2: hindpaw lifting and flinching) were calculated from total scores of 10 times per hindpaw (Abbott et al., 1995; Caspani et al., 2009; Draxler et al., 2014). To investigate the light-induced responses of animals, we were careful to establish experimental conditions before light stimuli. First, the animals were awake. Second, the extremities were on the plate without sticking to the wall. Thirds, the animals were at rest without moving or walking (Iyer et al., 2014).

### von Frey test

The method used for von Frey test were previously described (Tsuda et al., 2003). Briefly, calibrated von Frey filaments (0.4–15 g, Stoelting) were applied to the plantar surface of the rat hindpaw, and the 50% paw withdrawal threshold was determined.

### Intrathecal administration of morphine and pregabalin

A 32-gauge intrathecal catheter (ReCathCo) was inserted into the lumbar enlargement under isoflurane (2%) anesthesia as previously described (Tsuda et al., 2009). At 14 d post-PNI, W-TChR2V4 rats were injected with morphine (15 μg/10 µl) or pregabalin (10 μg/10 µl) once through the catheter.

### Immunohistochemistry

Rats were anesthetized with pentobarbital (100 mg/kg, i.p.) and perfused transcardially with 100 ml of PBS (137 mM NaCl, 2.7 mM KCl, 1.5 mM KH_2_PO_4_, and 8.1 mM NaH_2_PO_4_; pH 7.4), followed by 250 ml ice-cold 4% paraformaldehyde/PBS. L4 DRGs were removed, postfixed, and placed in 30% (w/v) sucrose solution for 24 h at 4°C. DRGs were sectioned into 15-µm sections using cryostat (Leica CM1100). The sections were incubated in blocking solution for 2 h and followed by the primary antibodies for Neurofilament 200 (NF200; 1:1000; catalog #N4142; Sigma-Aldrich), tropomysin-related kinase C (TrkC; 1:5000; catalog #AF1404; R&D Systems), calcitonin gene-related peptide (CGRP; 1:4000; catalog #T-4032; Peninsula Laboratories), TrkA (1:2000; catalog #AF1056; R&D Systems), transient receptor potential vanilloid subtype 1 (TRPV1; 1:200; catalog #sc-12498; Santa Cruz), and isolectin B4 (IB4) Alexa Fluor 568 (1:200; catalog #I21412; Thermo Fisher Scientific) for 48 h at 4°C. The sections were washed and incubated with secondary antibodies conjugated with Alexa Fluor 546 (1:1000, Invitrogen) for 3 h. The sections were then analyzed by a confocal microscope (LSM700, Zeiss). We analyzed the percentage of NF200^+^, TrkC^+^, CGRP^+^, TrkA^+^, TRPV^+^, and IB4^+^ neurons within ChR2^+^ (Venus) neurons in three sections of the DRG of each rat with LSM700 Imaging System. For the size-frequency analyses, the cross-sectional areas of ChR2^+^/NF200^+^ DRG neurons with clearly visible nuclei (ipsilateral, 371 cells; contralateral, 316 cells) was measured.

For c-Fos and phosphorylated extracellular signal-regulated protein kinase (pERK) immunostaining in the SDH, the W-TChR2V4 rats (day 14 post-PNI) were habituated for 15 min under anesthesia by isoflurane, and the plantar surface of the hindpaw of the rats was simulated for 10 min (c-Fos) and for 1 min (pERK) with blue light (duration, 500 ms; interval, 500 ms). At 1.5 h (c-Fos) and immediately (pERK) after stimulus, the rats were deeply anesthetized by isoflurane and fixed as describe above, and the L4 spinal cord was obtained. Transverse spinal cord (30 μm) and coronal brain (40 μm) sections were stained by primary antibodies [anti-c-Fos (rabbit polyclonal, 1:20,000; catalog #PC38; Millipore); anti-phospho-p44/p42 MAPK (pERK; rabbit polyclonal 1:500; catalog #9102; Cell Signaling); anti-neuronal nuclei (NeuN; mouse monoclonal, 1:2000; catalog #MAB377; Millipore Bioscience Research Reagents)]. For quantification, two to four sections from the L4 spinal cord segments were randomly selected from each rat, the number of c-Fos^+^ and pERK^+^ neurons with NeuN in the superficial lamina I-IIo was counted with ImageJ (http://rsbweb.nih.gov/ij/).

### Whole-cell patch-clamp recording

Under anesthesia with urethane (1.2–1.5 g/kg, i.p.), the lumbosacral spinal cord was removed from W-TChR2V4 rats and placed into a cold, high-sucrose, artificial CSF (ACSF; 250 mM sucrose, 2.5 mM KCl, 2 mM CaCl_2_, 2 mM MgCl_2_, 1.2 mM NaH_2_PO_4_, 25 mM NaHCO_3_, and 11 mM glucose). A parasagittal spinal cord slice (250–300 µm thick) with an attached L4 dorsal root was made using a vibrating microtome (VT1200, Leica), and the slices were maintained in oxygenated ACSF solution (125 mM NaCl, 2.5 mM KCl, 2 mM CaCl_2_, 1 mM MgCl_2_, 1.25 mM NaH_2_PO_4_, 26 mM NaHCO_3_, and 20 mM glucose) at room temperature (22–25°C) for at least 30 min. The spinal cord slice was then put into a recording chamber where it was continuously superfused with ACSF solution at 30–34°C at a flow rate of 4–7 ml/min. SDH neurons were visualized with an upright microscope equipped with infrared differential interference contrast Nomarski (FN1, Nikon). Lamina II was identified as a translucent band across the SDH, and for Lamina I neurons, only neurons at a distance of <30 μm from the border between the white and gray matter in the SDH were used (Prescott and De Koninck, 2002; Chen and Gu, 2005). The cellular location was confirmed by immunohistochemistry, in which cells were loaded with 0.4% Neurobiotin via a patch pipette (Yasaka et al., 2007). The patch pipettes were filled with an internal solution (125 mM K-gluconate, 10 mM KCl, 0.5 mM EGTA, 10 mM HEPES, 4 mM ATP-Mg, 0.3 mM NaGTP, 10 mM phosphocreatine, and 0.4% Neurobiotin; pH 7.28 adjusted with KOH). The pipette tip resistance was 4–7 MΩ. Synaptic currents were recorded using a computer-controlled amplifier (Axopatch 700B, Molecular Devices). The data were digitized with an analog-to-digital converter (Digidata 1550), stored on a personal computer using a data acquisition program (pCLAMP 10.4 acquisition software), and analyzed using software package (Clampfit version 10.4). EPSCs were recorded in the voltage-clamp mode at a holding potential of –70 mV, and the dorsal roots were stimulated with a suction electrode (Yoshimura and Nishi, 1993). A monopolar electrode was placed on the proximal part of the dorsal root, and an optical fiber with a 200-µm tip width was placed between the two electrodes. Aβ, Aδ, or C afferent-mediated responses were distinguished on the basis of conduction velocity (C < 0.8 m/s; Aδ, 2–11 m/s), as previously described (Kato et al., 2004). Aβ/δ and C fiber-evoked EPSCs were considered to be monosynaptic responses if the latency remained constant when the root was stimulated at 20 Hz for the Aβ/δ fiber-evoked EPSCs, and there was no failure regardless of constancy of latency stimulated at 2 Hz for C fiber-evoked EPSCs, respectively (Nakatsuka et al., 2000). Light-evoked EPSCs were considered to be monosynaptic responses if they did not exhibit failures on repetitive stimulation at 1 Hz and if they exhibited a low jitter on stimulation at 0.1 Hz (Honsek et al., 2015). The precise conduction velocity was determined by measuring the difference in response latencies evoked by the focal monopolar electrode and suction electrode (Baba et al., 1999; Kato et al., 2004).

### Place aversion

W-TChR2V4 rats were placed in a two-chambered box (45 cm width, 10 cm depth, 40 cm height) with black and white compartments and were allowed to explore the two chambers for 15 min. At first, the rat location was continuously monitored and analyzed for time spent in each of the chambers. This experiment ensured that the rats exhibited a preference for the black compartment. Blue light stimuli (wavelength, 470 nm; duration, 500 ms; interval, 500 ms) were delivered to the plantar skin of the left hindpaw of each rat with or without PNI, only when the testing rat was in the black compartment. The time spent in each compartment was measured for 20 min. Data were calculated by converting the amount of time spent in each compartment to the percentage of time spent in the white compartment.

### Silencing C fibers *in vivo*

C Fibers were pharmacologically silenced *in vivo* by previously reported methods (Binshtok et al., 2007). Under isoflurane (2%) anesthesia, W-TChR2V4 rats on 14 d post-PNI were intraplantarly injected with 10 µl of QX-314 (2%, Sigma-Aldrich) with vehicle (endotoxin-free water), QX-314 (2%) with capsaicin (10 µg; Sigma-Aldrich). Behavioral measurements were conducted before, 30, 60, and 90 min after the drug administration.

### Statistical analysis

Statistical analyses of the results were conducted with one-way repeated ANOVA with a *post hoc* Dunnett’s test, one-way repeated ANOVA with a *post hoc* Tukey’s test, paired Student’s *t* test, Mann–Whitney test and unpaired *t* test with Welch’s correction using GraphPad Prism 4.03 and GraphPad Prism 7.01 software. Statistical significance was set at *p* < 0.05.

## Results

### Pain-like behaviors by light illumination in W-TChR2V4 rats after PNI

**Figure 1.**
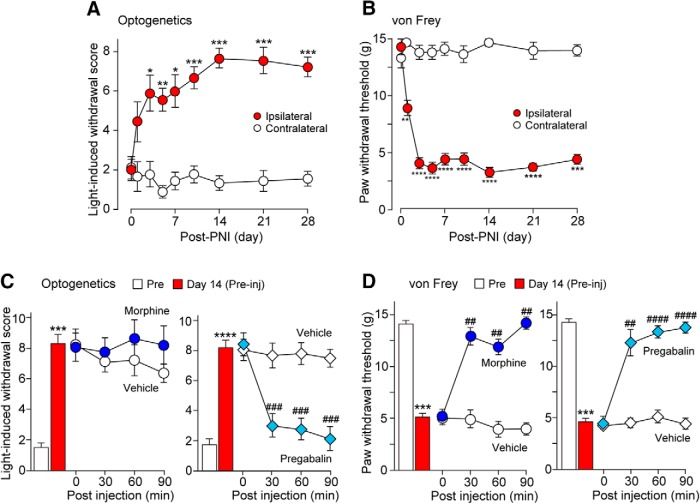
Optogenetically stimulated Aβ fibers induce pain-like behaviors after PNI. ***A***, ***B***, Withdrawal score by light (***A***) and paw withdrawal threshold by von Frey filaments (***B***) of W-TChR2V4 rats before and after PNI (*n* = 9 rats; ****p < 0.0001, ***p < 0.001, **p < 0.01, *p < 0.05 vs value on day 0, repeated-measures one-way ANOVA with *post hoc* Dunnett’s test). ***C***, Effect of intrathecal morphine (15 µg; left) or pregabalin (10 µg; right) on light-induced withdrawal responses of W-TChR2V4 rats on 14 d post-PNI [*n* = 7 rats; ****p < 0.0001, ***p < 0.001 vs value before PNI (Pre), paired *t* test; ###*p* < 0.001 vs value at 0 min, repeated-measures one-way ANOVA with *post hoc* Dunnett’s test]. ***D***, Paw withdrawal threshold of W-TChR2V4 rats in von Frey testing on 14 d post-PNI before and after intrathecal morphine (15 µg; left) or pregabalin [10 µg; right; *n* = 6 rats, ***p < 0.001 vs value before PNI (Pre), paired *t* test; ####*p* < 0.0001, ##*p* < 0.01 vs value at 0 min, repeated-measures one-way ANOVA with *post hoc* Dunnett’s test]. Values represent mean ± SEM.

To determine whether optogenetic stimulation of non-nociceptive myelinated A fibers results in pain-like behaviors after PNI, we applied blue laser light illumination to the plantar skin of the hindpaw of W-TChR2V4 rats before and after PNI. On day 14 post-PNI, light illumination to the plantar skin of the contralateral hindpaw produced no reaction or only mild movement without any lifting or flinching behaviors (Movie 1). However, illumination to the plantar skin of the hindpaw ipsilateral to PNI resulted in lifting and flinching behaviors. The total score of light-induced withdrawal behaviors in the ipsilateral side gradually increased, peaked at day 14 post-PNI, and continued until the final tested time point [Fig. 1*A*; *n* = 9 rats; ***p < 0.001, **p < 0.01, *p < 0.05 vs value on day 0, repeated-measures one-way ANOVA (*F*_(4.118,32.94)_ = 8.8, *p* < 0.0001) with *post hoc* Dunnett’s test]. In contrast, the withdrawal score of the contralateral hindpaw remained unchanged. In addition, we confirmed in von Frey testing that these rats also showed mechanical hypersensitivity to the filaments in the ipsilateral hindpaw after PNI [Fig. 1*B*; *n* = 9 rats; ****p < 0.0001, ***p < 0.001, **p < 0.01 vs value on day 0, repeated-measures one-way ANOVA (*F*_(4.001,32.01)_ = 55.63, *p* < 0.0001) with *post hoc* Dunnett’s test]. These findings indicate that blue light illumination to the plantar skin of the hindpaw of W-TChR2V4 rats with PNI evokes pain-like withdrawal responses.

Movie 1. Behavioral response evoked by light illumination to the hindpaw of W-TChR2V4 rat 14 d post-PNI. The plantar skin of the hindpaw was illuminated with a blue light emitting diode light (wavelength, 470 nm; duration, 500 ms). On day 14 post-PNI, light illumination to the plantar skin of the contralateral hindpaw in the W-TChR2V4 rat produced no reaction, but illumination to the ipsilateral plantar skin resulted in lifting and flinching behaviors. 10.1523/ENEURO.0450-17.2018.video.1

We next evaluated the sensitivity of two major analgesics (morphine and pregabalin) to these behaviors. We found that the light-induced withdrawal behaviors in W-TChR2V4 rats were not suppressed by intrathecal morphine (15 µg) but efficiently reversed by pregabalin [10 µg; Fig. 1*C*; *n* = 7 rats; ****p < 0.0001, ***p < 0.001 vs value before PNI (Pre), paired *t* test (morphine, *t* = 6.165, df = 6, *p* = 0.0008; pregabalin, *t* = 10.76, df = 7, *p* < 0.0001); ####*p* < 0.0001, ###*p* < 0.001 vs value at 0 min, repeated-measures one-way ANOVA (morphine, *F*_(1.596,9.576)_ = 0.2892, *p* = 0.7076; pregabalin, *F*_(2.641,18.49)_ = 35.39, *p* < 0.0001) with *post hoc* Dunnett’s test]. Systemic administration of morphine (3 mg/kg) was also ineffective on withdrawal responses by light (data not shown). In contrast, both intrathecal administration of morphine and pregabalin in W-TChR2V4 rats on day 14 post-PNI significantly elevated paw withdrawal threshold in von Frey testing [Fig. 1*D*; *n* = 6 rats, ***p < 0.001 vs value before PNI (Pre), paired *t* test (morphine, *t* = 9.836, df = 5, *p* = 0.0002; pregabalin, *t* = 10.76, df = 7, *p* = 0.0002); ####*p* < 0.0001, ##*p* < 0.01 vs value at 0 min, repeated-measures one-way ANOVA (morphine, *F*_(2.876,14.38)_ = 21.49, *p* < 0.0001; pregabalin, *F*_(1.853,11.12)_ = 31.02, *p* < 0.0001) with *post hoc* Dunnett’s test]. Suppression of withdrawal behavior in von Frey testing by morphine was consistent with previously reported results (Gao et al., 2004; Cheng et al., 2017) . Locomotor activities of PNI rats remained unchanged following intrathecal administration of pregabalin (10 µg; vehicle, 1.36 ± 0.35 m/min, *n* = 9; pregabalin, 1.37 ± 0.49 m/min, *n* = 8). These results indicate that the light-elicited withdrawal responses in PNI rats are morphine-resistant, but pregabalin-controllable pain-like behaviors.

### ChR2 expression in primary afferent Aβ fibers

**Figure 2.**
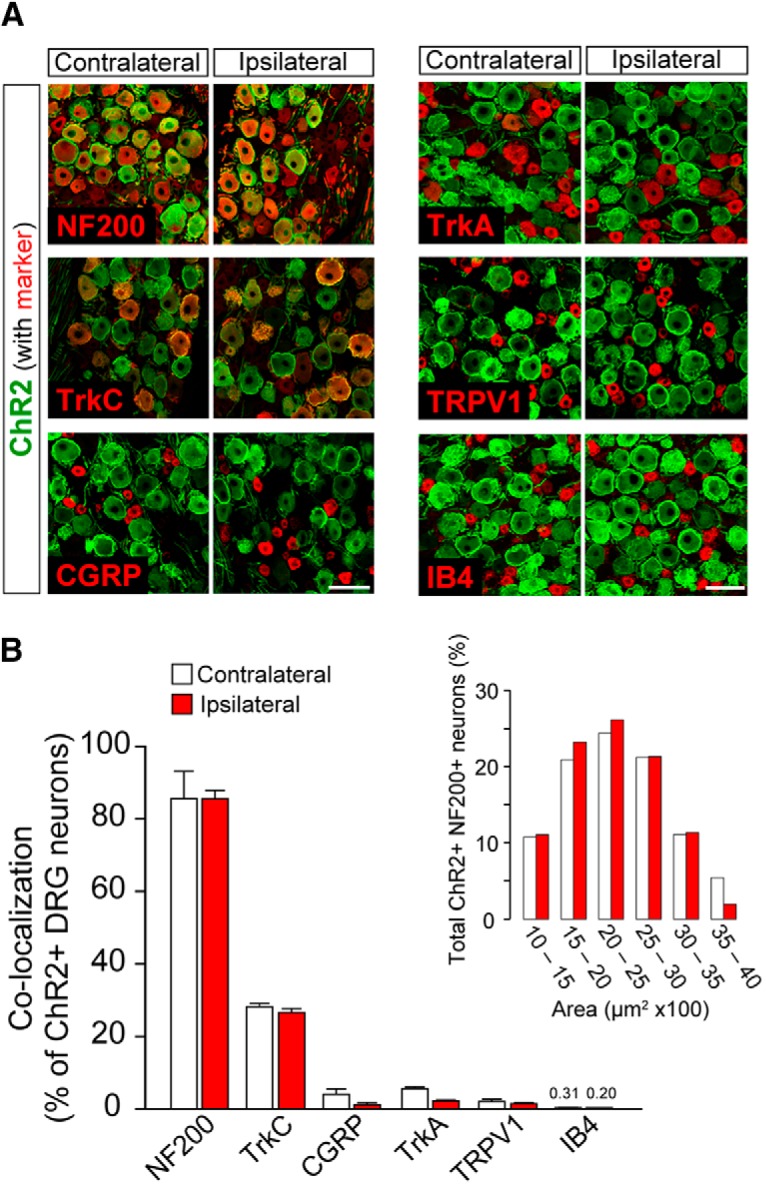
Immunohistochemical characterization of ChR2-expressing DRG neurons. ***A***, Immunolabeling of ChR2^+^ neurons (Venus; green) with either NF200, TrkC, CGRP, TrkA, TRPV1, or IB4 (red) in the L4 DRG contralateral and ipsilateral to PNI on day 14. ***B***, Percentage of colocalization of each marker in ChR2^+^ DRG neurons [*n* = 4 rats (two to three slices per rat)]. Size-frequency histogram (inset) illustrating the distribution of the cross-sectional areas of ChR2^+^ NF200^+^ DRG neurons in the L4 DRG ipsilateral (371 cells) and contralateral (316 cells) to PNI on day 14. Values represent mean ± SEM. Scale bar: 100 μm.

In W-TChR2V4 rats, ChR2 is selectively expressed in innocuous mechanoreceptive A fibers, including Aβ (Ji et al., 2012). However, PNI has been reported to change gene expression in spared DRG neurons (Fukuoka et al., 2002). To precisely analyze the immunohistochemnical characteristic of ChR2^+^ DRG neurons after PNI, we used six markers and examined ChR2^+^ neurons in the L4 DRG, whose afferents were uninjured and might convey sensory information from the hindpaw in the PNI model. Similar to the contralateral side of L4 DRG neurons, ChR2^+^ neurons in the ipsilateral side was highly coexpressed NF200 (Fig. 2*A*), a marker of myelinated Aβ and Aα fibers (and also, to a lesser extent, Aδ fibers; Usoskin et al., 2015). The percentage [Fig. 2*B*; *n* = 4 rats (two to three slices per rat)] and the size-frequency [Fig. 2*B*, inset; in the L4 DRG ipsilateral (371 cells) and contralateral (316 cells) to PNI on day 14] of ChR2^+^ DRG neurons expressing NF200 were almost identical between ipsilateral and contralateral sides. This study also found that ChR2^+^ DRG neurons express TrkC (Fig. 2*A*), which is a marker of low-threshold mechanoreceptors and proprioceptors (Usoskin et al., 2015), the percentages of which was also indistinguishable between ipsilateral and contralateral sides (Fig. 2*B*). In addition, there were few ChR2^+^ DRG neurons double-labeled with either TRPV1 (a marker of nociceptors), CGRP (a marker of peptidergic C fibers and partly of Aδ fibers), TrkA (a marker of peptidergic C fibers and nociceptive A fibers including Aβ), or IB4 (a marker of non-peptidergic C fibers) in the DRG ipsilateral and contralateral to the PNI (Fig. 2*A*,*B*). To exclude a possible involvement of ChR2^+^ TPRV1^+^ DRG neurons in the light-elicited pain-like withdrawal responses, we tested the effect of functional silencing of TRPV1^+^ DRG neurons by intraplantar coadministration of the lidocaine derivative QX-314 and capsaicin (or its vehicle as controls; Binshtok et al., 2007; Shen et al., 2012). On day 14 post-PNI, intraplantar QX-314 and capsaicin treatment did not suppress the PNI-induced increase in withdrawal scores [mean ± SEM; QX314 + vehicle (*n* = 8), 7.5 ± 0.5 at preinjection, 7.5 ± 0.5, 6.8 ± 0.7, and 7.0 ± 0.4 at 30, 60, and 90 min postinjection, respectively; QX314 + capsaicin (*n* = 8), 8.1 ± 0.9 at preinjection, 8.4 ± 0.8, 7.5 ± 0.8, and 6.8 ± 0.8 at 30, 60, and 90 min postinjection, respectively]. This result suggests little, if any, contribution of ChR2^+^ TPRV1^+^ DRG neurons.

### Stimulation of primary afferent Aβ fibers by light

**Figure 3.**
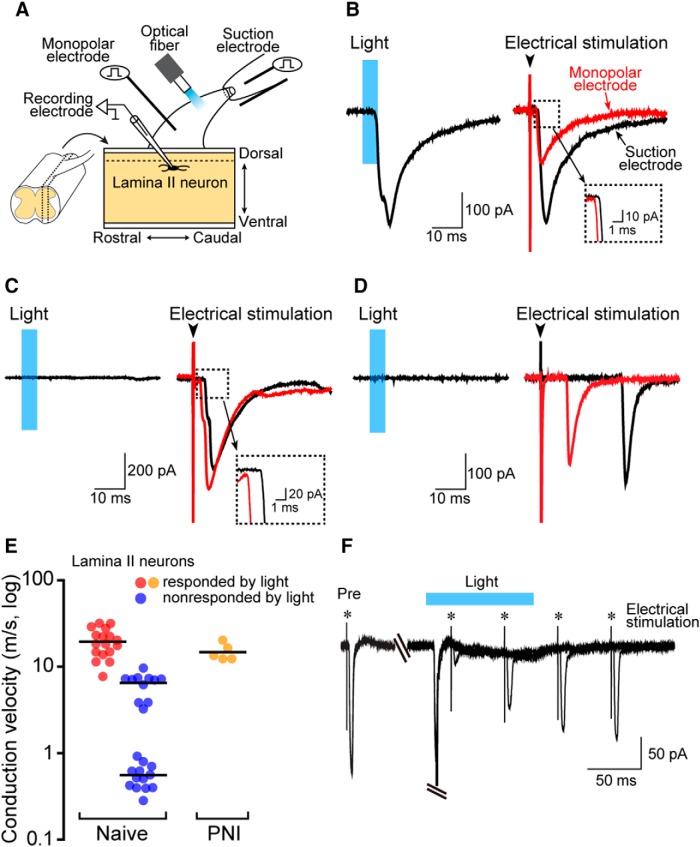
Light illumination activates primary afferents with conduction velocity in a range of Aβ fibers. ***A***, Schematic diagram of whole-cell patch-clamp recording in SDH Lamina II neurons using sagittal spinal cord slices with the L4 dorsal root of W-TChR2V4 rats. A suction electrode was used to electrically stimulate the dorsal root. A monopolar stimulating electrode for calculating conduction velocity was placed at the proximal point. Optical fiber was placed between two electrodes. ***B***–***D***, Representative averaged traces of EPSCs evoked by light (left) and electrical (right) stimulation recorded from Lamina II SDH neurons. The averaged traces of EPSCs recorded from the same neuron evoked by suction electrode (distal, black line) and monopolar electrode placed in proximal dorsal root (proximal, red line). ***E***, Conduction velocity (m/s) of primary afferent fibers responded and non-responded by light illumination. Calculated conduction velocity of fibers with monosynaptic input to a recorded neuron was indicated by a single circle [red, responded neurons of naive rats, *n* = 17; blue, non-responded neurons of naive rats, *n* = 24; orange, responded neurons of PNI rats (day 14), *n* = 5]. ***F***, Effects of light illumination on monosynaptic Aβ fiber-mediated EPSCs evoked by electrical stimulation (monopolar electrode) of the dorsal root in Lamina II neurons. The dorsal root was electrically stimulated before (Pre) and at 20, 70, 120, and 170 ms after light illumination for 100 ms. Similar results were seen in each of two experiments. Blue regions, light illumination to the dorsal roots. Values represent mean ± SEM.

To functionally assess whether Aβ fibers are selectivity activated by light illumination, we performed whole-cell patch-clamp recordings in Lamina II SDH neurons using spinal cord slices with the L4 dorsal root of W-TChR2V4 rats (Fig. 3*A*) because in this lamina there are neurons that receive monosynaptic inputs from either Aβ, Aδ, or C fibers. In Lamina II neurons receiving excitatory monosynaptic inputs by light from optical fiber and electrical stimulation of the dorsal root from a suction electrode (Fig. 3*B*), we found monosynaptic EPSCs evoked by electrical stimulation of the dorsal root from a monopolar electrode (Fig. 3*B*) placed at the proximal point (Fig. 3*A*) with a faster latency than EPSCs by electrical stimulation from the suction electrode (Fig. 3*B*, inset). We then calculated the conduction velocity by the latencies of monosynaptic EPSCs evoked by electrical stimulation at the two different places (suction and monopolar electrodes; Kato et al., 2004) and found that the conduction velocity of these fibers was 19.5 ± 1.7 m/s (*n* = 17; Fig. 3*E*, red circles), which was in the range of conduction velocity of Aβ fibers (Nakatsuka et al., 1999). The Aβ fiber-mediated EPSCs by the electrical stimulation in Lamina II neurons were markedly decreased by the pre-stimulation of the root with light (conduction block; Fig. 3*F*), implying that light activates Aβ fibers. On the other hand, the latencies of monosynaptic EPSCs by electrical stimulations in Lamina II neurons non-responded by light were clearly distinct from those of neurons responded by light (Fig. 3*C*,*D*), and the calculated conduction velocity was divided into two groups (Fig. 3*E*): the faster group was 6.2 ± 0.6 m/s (*n* = 11), and the slower groups was 0.54 ± 0.05 m/s (*n* = 13; Fig. 3*E*, blue circles), which were in the range of conduction velocity of Aδ and C fibers, respectively (Nakatsuka et al., 1999). Furthermore, we confirmed that the conduction velocity of light-responding primary afferents was not changed by PNI (Fig. 3*E*). Collectively, these immunohistochemical and electrophysiological experiments all support excitation of non-nociceptive Aβ fibers by light.

### Excitation of Lamina I SDH neurons by optogenetic Aβ fiber stimulation after PNI

**Figure 4.**
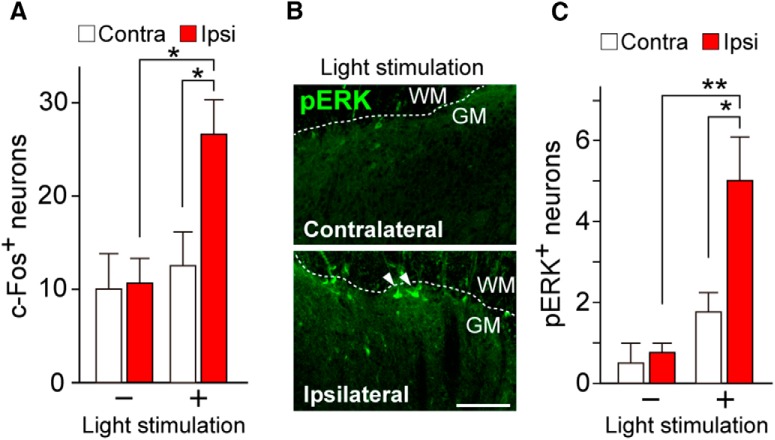
Induction of c-Fos and pERK in SDH neurons by light after PNI. ***A***, Number of c-Fos^+^ neurons in the superficial L4 SDH of W-TChR2V4 rats with or without light stimulation at day 14 post-PNI [*n* = 4 rats (two to four slices per rat); *p < 0.05, one-way ANOVA with *post hoc* Tukey’s test]. ***B***, ***C***, Immunofluorescence (***B***) and number (***C***) of pERK^+^ neurons in the superficial L4 SDH of W-TChR2V4 rats with or without light stimulation at day 14 post-PNI. WM, white matter; GM, gray matter [*n* = 4–5 rats (three slices per rat); **p < 0.01, *p < 0.05, one-way ANOVA with *post hoc* Tukey’s test]. Values represent mean ± SEM. Scale bar: 100 µm.

We further investigated input of optogenetically evoked signals to the SDH. In immunohistochemistry, using the neuronal activity marker c-Fos, we observed that the number of c-Fos^+^ cells in the superficial SDH significantly increased following light illumination to the hindpaw of W-TChR2V4 rats at 14 d post-PNI [Fig. 4*A*; *n* = 4 rats (two to four slices per rat); *p < 0.05, one-way ANOVA (*F*_(3,12)_ = 6.548, *p* = 0.0072) with *post hoc* Tukey’s test]. These c-Fos^+^ cells colocalized with the neuronal marker NeuN (data not shown). Consistent with behavioral data, intrathecal morphine had no effect on the light-induced c-Fos^+^ neurons, but intrathecal pregabalin significantly reduced the number of c-Fos^+^ neurons (vehicle, 26.4 ± 0.4, *n* = 3 rats; morphine, 24.0 ± 2.1, *n* = 5 rats; pregabalin, 13.5 ± 1.0, *n* = 4 rats; *p* < 0.01). We then examined pERK, a marker of nociceptive-specific activation of SDH neurons (Ji et al., 1999). In the contralateral side, there was no change in the number of pERK^+^ SDH neurons with light stimulation compared with no light stimulation (Fig. 4*B*,*C*). However, in PNI rats, pERK^+^ neurons appeared in superficial SDH by light illumination (Fig. 4*B*), and there was a clear increase in the number of pERK^+^ neurons in the superficial lamina by light stimulation [Fig. 4*C*; *n* = 4–5 rats (three slices per rat); **p < 0.01, *p < 0.05, one-way ANOVA (*F*_(3,13)_ = 11.27, *p* = 0.0006) with *post hoc* Tukey’s test]. In PNI rats without light stimulation, the number of pERK^+^ cells slightly, but not significantly, increased.

**Figure 5.**
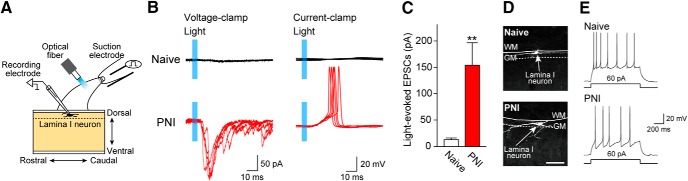
Light illumination excites Lamina I SDH neurons after PNI. ***A***, Schematic diagram of whole-cell patch-clamp recording in Lamina I neurons using sagittal spinal cord slices with the L4 dorsal root taken from W-TChR2V4 rats with or without PNI (day 14). ***B***, Representative traces of EPSCs (voltage-clamp mode; left) and action potentials (current-clamp mode; left) in Lamina I neurons at the L4 in spinal slices taken from W-TChR2V4 rats (naive and PNI). Blue regions, light illumination to the dorsal roots. ***C***, Amplitude of light-evoked EPSCs in Lamina I neurons (naive and PNI; *n* = 10 cells; **p < 0.01, unpaired *t* test with Welch’s correction). ***D***, ***E***, Confocal images (***D***) and firing pattern (***E***) of recorded Lamina I neurons in W-TChR2V4 rats with or without PNI. Values represent mean ± SEM. Scale bar: 100 µm.

These results suggested that optically stimulated signals after PNI could be conveyed to Lamina I SDH neurons. To directly determine this, we performed whole-cell patch-clamp recordings using spinal cord slices of W-TChR2V4 rats and measured synaptic activity of Lamina I neurons evoked by blue light stimulation to the dorsal roots (Fig. 5*A*). In SDH slices of naive rats, light stimulation did not evoke EPSCs in Lamina I neurons (Fig. 5*B*, upper right traces) or produced little, if any, EPSCs in some Lamina I neurons. We excluded the possibility that the recorded neurons were dysfunctional, because electrical stimulation of the dorsal roots evoked C or Aδ fiber-EPSCs (data not shown) in all tested neurons. These results suggested that Lamina I neurons in naive rats did not receive optogenetically evoked excitatory signals. However, in slices from PNI rats, nine of ten Lamina I SDH neurons elicited profound polysynaptic EPSCs following light illumination (Fig. 5*B*, bottom left traces). The amplitudes of light-evoked EPSCs significantly increased after PNI [Fig. 5*C*; *n* = 10 cells; **p < 0.01, unpaired *t* test (*t* = 3.314, df = 9, *p* = 0.0090) with Welch’s correction]. Moreover, light illumination induced five Lamina I neurons to fire action potentials at a resting membrane potential (Fig. 5*B*, bottom right traces). The recorded neurons in spinal slices of naive and PNI rats exhibited a morphology with rostro-caudally extended dendrites (Fig. 5*D*), and a depolarizing current injection at the resting membrane potential resulted in a tonic discharge (Fig. 5*E*), both of which have been reported in nociceptive projection neurons in Lamina I (Prescott and De Koninck, 2002; Ruscheweyh and Sandkühler, 2002; Ruscheweyh et al., 2004; Lolignier et al., 2015). Together, these findings indicate that optogenetic stimulation of Aβ fibers following PNI results in excitation of Lamina I SDH neurons (presumably nociceptive).

### Aversive behavioral symptom induced by light after PNI

**Figure 6.**
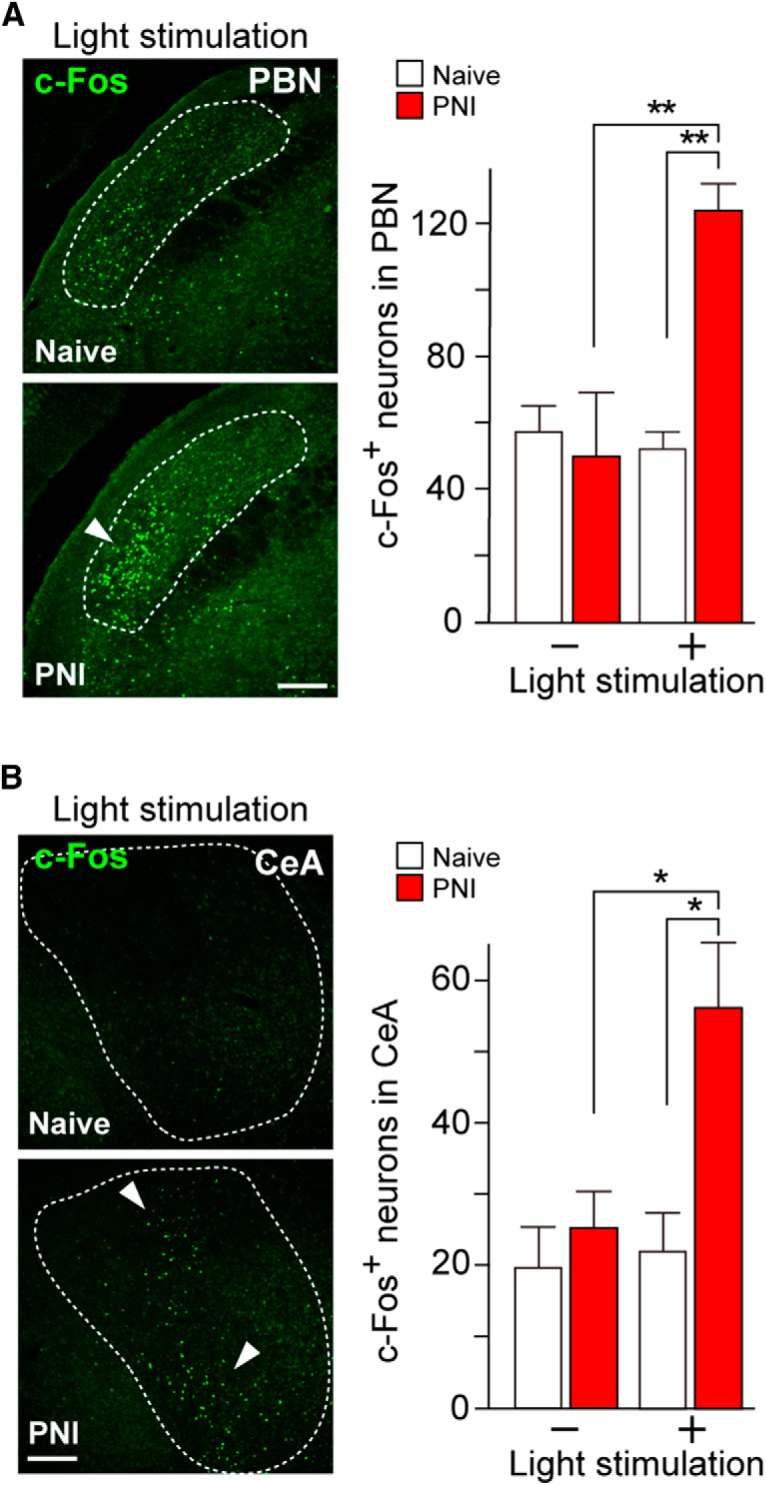
Activation of PBN and CeA by illuminating the hindpaw after PNI. ***A***, ***B***, Immunofluorescence (left) and number (right) of c-Fos^+^ neurons in the PBN (***A***) and CeA (***B***) of W-TChR2V4 rats [naive and PNI (day 14)] with or without light stimulation [*n* = 3–4 rats (three slices per rat); **p < 0.01, *p < 0.05, one-way ANOVA with *post hoc* Tukey’s test). Values represent mean ± SEM. Scale bar: 200 µm.

Because the majority of lamina I SDH neurons provide ascending signals to the parabrachial nucleus (PBN; Hunt and Mantyh, 2001; Todd, 2010), we quantified c-Fos^+^ neurons in the PBN of W-TChR2V4 rats following PNI. Results showed that light stimulation increased the number of c-Fos^+^ cells in the side corresponding to the light-illuminated hindpaw [Fig. 6*A*; *n* = 3–4 rats (three slices per rat); **p < 0.01, one-way ANOVA (*F*_(3,13)_ = 8.918, *p* = 0.0028) with *post hoc* Tukey’s test]. Light-induced c-Fos expression was also observed in the central nucleus of the amygdala [CeA; Fig. 6*B*; *n* = 3–4 rats (three slices per rat); *p < 0.05, one-way ANOVA (*F*_(3,10)_ = 7.008, *p* = 0.0081) with *post hoc* Tukey’s test], which forms connections with the PBN (Basbaum et al., 2009; Draxler et al., 2014; Sugimura et al., 2016). In W-TChR2V4 rats without PNI, the number of c-Fos^+^ cells remained unchanged in both regions with or without light stimulation (Fig. 6*A*,*B*). Thus, light stimulation after PNI may elicit activation of the SDH-PBN-CeA pain pathway.

**Figure 7.**
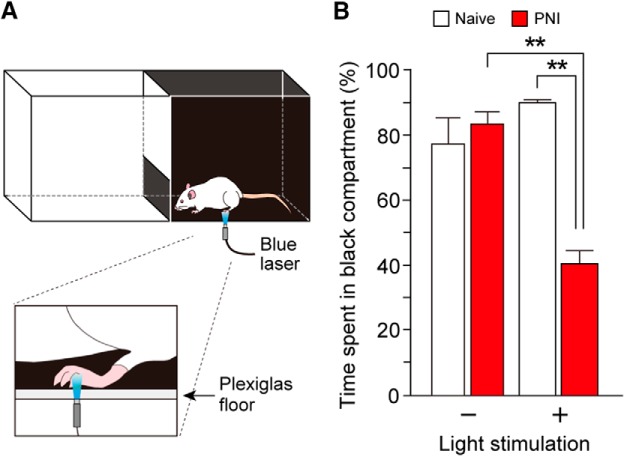
Aversive behavioral responses induced by light illumination after PNI. ***A***, Schematic diagram for place aversion test. Rat received blue light to the plantar skin while in the black compartment. ***B***, Percentage of time spent in black compartment [naive and PNI (day 14)] with or without light stimulation (*n* = 5–7 rats; **p < 0.01, one-way ANOVA with *post hoc* Tukey’s test). Values represent mean ± SEM.

Pain has both sensory and emotional aspects. The CeA is considered to be the core brain region that processes aversive information of the pain experience (Basbaum et al., 2009; Draxler et al., 2014). Therefore, we examined whether W-TChR2V4 rats with PNI display an aversion to an area where light illumination was applied to their hindpaw. We constructed a place-aversion apparatus that comprised two equal-sized compartments with distinct visual cues (one was black, and the other was white) with a clear Plexiglas floor (Fig. 7*A*). We applied light to the hindpaw only when the testing rat was in the black compartment. Under our experimental condition, where normal rats exhibited an ∼80% preference for the black compartment, the preference remained unchanged with blue light illumination (Fig. 7*B*). After PNI, rats without light illumination exhibited a similar preference for the black compartment. However, when the hindpaw was illuminated while the rats were in the black compartment, they displayed a significant decrease in time spent in the illuminated black compartment [Fig. 7*B*; *n* = 5–7 rats; **p < 0.01, one-way ANOVA (*F*_(3,20)_ = 8.692, *p* = 0.0007) with *post hoc* Tukey’s test]. These results suggest that PNI rats exhibit an aversion to light illumination.

## Discussion

This study demonstrates for the first time that optogenetic stimulation of non-nociceptive Aβ fibers after PNI produces neuropathic pain-like behaviors with sensory and aversive emotional aspects in awake, freely moving W-TChR2V4 rats. In the DRG of the W-TChR2V4 rat line, ChR2 was selectively expressed in neurons with markers of myelinated, low-threshold mechanoreceptors (NF200 or TrkC), but not in neurons with markers of nociceptors. The percentage of ChR2^+^ DRG (L4) neurons colocalized with each marker was not changed by PNI. ChR2 in the skin of W-TChR2V4 rats have been shown to be expressed at nerve endings of myelinated primary afferents that are associated with Merkel cells and lamellar cells to form Meissner’s corpuscles-like structures (Ji et al., 2012). Our electrophysiological data in the conduction velocity of optically stimulated afferents and the conduction block also strongly suggest a selective activation of Aβ fibers by blue light illumination under normal and PNI conditions. It has been reported that there is an Aβ fiber subgroup characterized as nociceptors in which TrkA is highly expressed (Fang et al., 2005). However, we showed that few ChR2^+^ DRG neurons expressed TrkA under normal and PNI conditions. The W-TChR2V4 line has been found in several transgenic rat lines in which ChR2 transgene is driven by the Thy-1.2 promoter. It has been reported that Thy-1 in the mouse DRG is expressed not only in low-threshold mechanoreceptors but also in peptidergic nociceptors expressing CGRP (Usoskin et al., 2015). However, consistent with the previous data (Ji et al., 2012), we also confirmed that few ChR2^+^ DRG neurons were double-labeled by CGRP. In addition, in TRPV1^+^ DRG neurons in W-TChR2V4 rats, there was a small percentage (<2%) of ChR2^+^ neurons, but we found that functional silencing of TRPV1^+^ DRG neurons (Binshtok et al., 2007) had no effect on the light-evoked withdrawal behaviors after PNI, suggesting little, if any, contribution of ChR2^+^ TPRV1^+^ DRG neurons. A recent study reported that optogenetic stimulation of keratinocytes in the skin produces withdrawal behaviors (Baumbauer et al., 2015), but in W-TChR2V4 rats, ChR2 was not expressed in keratinocytes under normal (Ji et al., 2012) and PNI conditions (data not shown). Therefore, our findings suggest that illuminating blue-light to the plantar skin of W-TChR2V4 rats stimulates non-nociceptive Aβ fibers, which, after PNI, leads to production of neuropathic pain-like behaviors.

Our immunohistochemical and electrophysiological findings in the SDH revealed that optically generated Aβ fiber signals is conveyed to the SDH and excite Lamina I neurons after PNI. In fact, these SDH neurons in rats with PNI induced expression of the nociceptive marker pERK, and elicited polysynaptic EPSCs and action potentials by light. These electrophysiologically recorded neurons exhibited tonic discharges by depolarization and rostro-caudally extended dendrites, both of which have been previously reported in Lamina I projecting neurons. Furthermore, light illumination after PNI induced c-Fos expression in the PBN, where the majority of Lamina I nociceptive neurons project (Todd, 2010), and Aβ fiber-related stimuli (brush and touch) to the skin of PNI rats has been shown to excite PBN-projecting Lamina I SDH neurons (Keller et al., 2007), supporting our notion that light-evoked Aβ fiber stimulation could excite Lamina I projecting neurons after PNI. In this study, how Aβ fibers pathologically signal to Lamina I neurons remains to be determined. Lamina I neurons are thought to polysynaptically link Aβ fibers via SDH interneurons, but are normally not activated by these fibers due to the feed-forward activation of inhibitory interneurons that gate Aβ signaling flow to Lamina I neurons (Sandkühler, 2009; Braz et al., 2014; Prescott et al., 2014; Peirs and Seal, 2016). Indeed, impaired function of inhibitory SDH interneurons (disinhibition) induces mechanical hypersensitivity in von Frey testing (Duan et al., 2014; Foster et al., 2015; Petitjean et al., 2015). In addition, it is possible that PNI increases a neural connection of Aβ fibers onto excitatory interneurons in Lamina II whose activation leads to excitation of Lamina I neurons. Therefore, in further investigations using our optogenetic approach, it will be important to determine if such disinhibition is involved in the Aβ fiber-induced excitation of Lamina I neurons and neuropathic pain-like behaviors after PNI, which would be a crucial step for identifying a polysynaptic circuit that Aβ fiber inputs pathologically relay to Lamina I neurons, converting non-nociceptive signals to nociceptive ones.

Pain has both sensory and affective dimensions. However, there is no study that has clearly detected an aversive response evoked by Aβ fibers in neuropathic pain models. This study demonstrated for the first time aversive behavioral symptoms induced by light-induced Aβ fiber stimulation after PNI, implying that optogenetically stimulated Aβ fibers induce not only sensory, but also emotional, components of pain. Because aversive information of the pain experience is processed in the CeA (Basbaum et al., 2009; Draxler et al., 2014) and c-Fos expression in the CeA was induced by light in the PNI rats, the CeA may be involved in the light-induced aversive behaviors. Furthermore, the CeA is functionally connected with the PBN (Basbaum et al., 2009; Draxler et al., 2014; Sugimura et al., 2016), and, interestingly, PNI produces potentiation of postsynaptic currents in CeA neurons evoked by electrical stimulation of PBN (Ikeda et al., 2007), indicating synaptic plasticity in the CeA under a neuropathic pain condition. Collectively, it is conceivable that non-nociceptive Aβ fibers in PNI rats activate the SDH–PBN–CeA pain pathway and produce aversion, although neural networks involving other brain regions to activate PBN or CeA neurons will be an important subject in future works. In addition, as W-TChR2V4 rats express ChR2 in retinal ganglion cells (Tomita et al., 2009), we cannot completely exclude a possible involvement of retinal ganglion cells in the light-induced aversive response after PNI, although light illumination to the hindpaw of normal W-TChR2V4 rats did not produce aversion and we focally illuminated to the hindpaw to minimize light diffusion.

Despite reversal of the paw withdrawal threshold by intrathecal morphine in von Frey testing, the light-induced neuropathic pain behaviors were resistant to morphine. This behavioral data were consistent with the failure of morphine to reduce c-Fos expression in SDH neurons following light illumination (our study) and brushing (Miraucourt et al., 2009). The mechanism underlying the ineffectiveness of morphine remains unclear and needs further investigations, but this may be explained by the Aβ-mediated pathway, because morphine does not inhibit Aβ fiber-evoked responses in the SDH (Le Bars et al., 1979; Dickenson and Sullivan, 1986) and μ-opioid receptors are mainly located on nociceptive fibers (Arvidsson et al., 1995; Basbaum et al., 2009). The pharmacologically different profile of morphine has also been reported in two distinct types of mechanical hypersensitivities, static and dynamic, which have been observed in neuropathic pain patients (Colloca et al., 2017) and animal models (Field et al., 1999; Cheng et al., 2017). Although static allodynia can be evoked by punctate stimuli like von Frey filaments and is sensitive to morphine, dynamic allodynia can be evoked by movement across the skin (e.g., stroking the skin with a soft brush) and is resistant to morphine (Field et al., 1999; Miraucourt et al., 2009; Cheng et al., 2017). Aβ fibers and small diameter nociceptive fibers have been implicated in dynamic and static allodynia, respectively (Ochoa and Yarnitsky, 1993; Yamamoto et al., 2008). There are similarities between light-induced pain behaviors and dynamic mechanical allodynia after PNI in terms of morphine resistance, Aβ fiber contribution, and pregabalin effectiveness. Because optogenetically activated Aβ fibers are innocuous mechanoreceptive fibers (Ji et al., 2012), pain-like behaviors evoked by light after PNI may mimic a sensory component of dynamic mechanical allodynia. However, we cannot exclude the possible involvement of other primary afferent subpopulations, such as low-threshold mechanoreceptive C and Aδ fibers (Li et al., 2011; Liljencrantz and Olausson, 2014; François et al., 2015), in dynamic allodynia.

One advantage of this method was the assessment of Aβ fiber-derived neuropathic pain behaviors in awake, freely moving animals without direct contact of the light probe to the skin, which is in stark contrast to tests using von Frey filaments, brushes, and electrical stimuli, and also without extensive training required for the previous methods. Our approach is methodologically much easier and is expected to improve the accuracy and reproducibility between investigators. Light illumination could also enable stimulation of the ChR2^+^ Aβ fibers in *in vitro* and *in vivo* experimental conditions. A technical limitation of previous methods using filaments and brushes is the inability of mechanical stimuli to activate afferent subpopulations in *in vitro* studies, such as electrophysiological experiments using spinal slices with dorsal roots. Thus, our model may aid future *in vivo* and *in vitro* studies to elucidate the mechanistic underpinnings for Aβ fiber-evoked neuropathic pain. This method can also concomitantly measure sensory and emotional components of neuropathic pain derived by Aβ fibers, suggesting that this model is clinically and translationally relevant for neuropathic pain.

In conclusion, we provide the first evidence that optogenetic activation of non-nociceptive Aβ fibers in freely moving animals produced neuropathic pain-like behaviors that were resistant to morphine treatment. Morphine resistance may correlate with the clinical situation in which neuropathic pain patients are often refractory to treatment. After PNI, optogenetic Aβ fiber stimulation caused excitation of Lamina I SDH neurons that were normally silent by this stimulation. Moreover, the value of our model was substantiated by showing that the PNI rats exhibited an aversion to hindpaw illumination, as well as activation of central amygdaloid neurons. In W-TChR2V4 rats, ChR2 in the skin is expressed at sensory nerve endings associated with tactile end organs (Ji et al., 2012). Thus, this method could provide a new approach for investigating the mechanisms underlying neuropathic mechanical allodynia with sensory and emotional features and for developing new drugs to treat neuropathic pain.

## Synthesis

Reviewing Editor: Karen Davis, Krembil Research Institute and University of Toronto Decisions are customarily a result of the Reviewing Editor and the peer reviewers coming together and discussing their recommendations until a consensus is reached. When revisions are invited, a fact-based synthesis statement explaining their decision and outlining what is needed to prepare a revision will be listed below. The following reviewer(s) agreed to reveal their identity: Marco Martina

The authors have provided a thorough response to the previous review.

## References

[B1] Abbott FV, Franklin KB, Westbrook RF (1995) The formalin test: scoring properties of the first and second phases of the pain response in rats. Pain 60:91–102. 7715946 10.1016/0304-3959(94)00095-V

[B2] Abrahamsen B, Zhao J, Asante CO, Cendan CM, Marsh S, Martinez-Barbera JP, Nassar MA, Dickenson AH, Wood JN (2008) The cell and molecular basis of mechanical, cold, and inflammatory pain. Science 321:702–705. 10.1126/science.1156916 18669863

[B3] Arvidsson U, Riedl M, Chakrabarti S, Lee JH, Nakano AH, Dado RJ, Loh HH, Law PY, Wessendorf MW, Elde R (1995) Distribution and targeting of a mu-opioid receptor (MOR1) in brain and spinal cord. J Neurosci 15:3328–3341. 7751913 10.1523/JNEUROSCI.15-05-03328.1995 PMC6578209

[B4] Baba H, Doubell TP, Woolf CJ (1999) Peripheral inflammation facilitates Abeta fiber-mediated synaptic input to the substantia gelatinosa of the adult rat spinal cord. J Neurosci 19:859–867. 9880605 10.1523/JNEUROSCI.19-02-00859.1999 PMC6782212

[B5] Basbaum AI, Bautista DM, Scherrer G, Julius D (2009) Cellular and molecular mechanisms of pain. Cell 139:267–284. 10.1016/j.cell.2009.09.028 19837031 PMC2852643

[B6] Baumbauer KM, DeBerry JJ, Adelman PC, Miller RH, Hachisuka J, Lee KH, Ross SE, Koerber HR, Davis BM, Albers KM (2015) Keratinocytes can modulate and directly initiate nociceptive responses. Elife 4 10.7554/eLife.09674 PMC4576133 26329459

[B7] Binshtok AM, Bean BP, Woolf CJ (2007) Inhibition of nociceptors by TRPV1-mediated entry of impermeant sodium channel blockers. Nature 449:607–610. 10.1038/nature06191 17914397

[B8] Boada MD, Martin TJ, Peters CM, Hayashida K, Harris MH, Houle TT, Boyden ES, Eisenach JC, Ririe DG (2014) Fast-conducting mechanoreceptors contribute to withdrawal behavior in normal and nerve injured rats. Pain 155:2646–2655. 10.1016/j.pain.2014.09.030 25267211 PMC4374598

[B9] Braz J, Solorzano C, Wang X, Basbaum AI (2014) Transmitting pain and itch messages: a contemporary view of the spinal cord circuits that generate gate control. Neuron 82:522–536. 10.1016/j.neuron.2014.01.018 24811377 PMC4492533

[B10] Carr FB, Zachariou V (2014) Nociception and pain: lessons from optogenetics. Front Behav Neurosci 8:69. 10.3389/fnbeh.2014.00069 24723861 PMC3971183

[B11] Caspani O, Zurborg S, Labuz D, Heppenstall PA (2009) The contribution of TRPM8 and TRPA1 channels to cold allodynia and neuropathic pain. PLoS One 4:e7383. 10.1371/journal.pone.0007383 19812688 PMC2753652

[B12] Chen M, Gu JG (2005) A P2X receptor-mediated nociceptive afferent pathway to lamina I of the spinal cord. Mol Pain 1:4. 10.1186/1744-8069-1-4 15813988 PMC1074354

[B13] Cheng L, Duan B, Huang T, Zhang Y, Chen Y, Britz O, Garcia-Campmany L, Ren X, Vong L, Lowell BB, Goulding M, Wang Y, Ma Q (2017) Identification of spinal circuits involved in touch-evoked dynamic mechanical pain. Nat Neurosci 20:804–814. 10.1038/nn.4549 28436981 PMC5470641

[B14] Colloca L, Ludman T, Bouhassira D, Baron R, Dickenson AH, Yarnitsky D, Freeman R, Truini A, Attal N, Finnerup NB, Eccleston C, Kalso E, Bennett DL, Dworkin RH, Raja SN (2017) Neuropathic pain. Nat Rev Dis Primers 3:17002. 10.1038/nrdp.2017.2 28205574 PMC5371025

[B15] Daou I, Beaudry H, Ase AR, Wieskopf JS, Ribeiro-da-Silva A, Mogil JS, Seguela P (2016) Optogenetic silencing of Nav1.8-positive afferents alleviates inflammatory and neuropathic pain. eNeuro 3 10.1523/ENEURO.0140-15.2016 PMC4794527 27022626

[B16] Dickenson AH, Sullivan AF (1986) Electrophysiological studies on the effects of intrathecal morphine on nociceptive neurones in the rat dorsal horn. Pain 24:211–222. 3754322 10.1016/0304-3959(86)90044-8

[B17] Draxler P, Honsek SD, Forsthuber L, Hadschieff V, Sandkühler J (2014) VGluT3(+) primary afferents play distinct roles in mechanical and cold hypersensitivity depending on pain etiology. J Neurosci 34:12015–12028. 10.1523/JNEUROSCI.2157-14.2014 25186747 PMC6130698

[B18] Duan B, Cheng L, Bourane S, Britz O, Padilla C, Garcia-Campmany L, Krashes M, Knowlton W, Velasquez T, Ren X, Ross SE, Lowell BB, Wang Y, Goulding M, Ma Q (2014) Identification of spinal circuits transmitting and gating mechanical pain. Cell 159:1417–1432. 10.1016/j.cell.2014.11.003 25467445 PMC4258511

[B19] Fang X, Djouhri L, McMullan S, Berry C, Okuse K, Waxman SG, Lawson SN (2005) trkA is expressed in nociceptive neurons and influences electrophysiological properties via Nav1.8 expression in rapidly conducting nociceptors. J Neurosci 25:4868–4878. 10.1523/JNEUROSCI.0249-05.2005 15888662 PMC6724783

[B20] Field MJ, Bramwell S, Hughes J, Singh L (1999) Detection of static and dynamic components of mechanical allodynia in rat models of neuropathic pain: are they signalled by distinct primary sensory neurones? Pain 83:303–311. 10534603 10.1016/s0304-3959(99)00111-6

[B21] Foster E, Wildner H, Tudeau L, Haueter S, Ralvenius WT, Jegen M, Johannssen H, Hösli L, Haenraets K, Ghanem A, Conzelmann KK, Bösl M, Zeilhofer HU (2015) Targeted ablation, silencing, and activation establish glycinergic dorsal horn neurons as key components of a spinal gate for pain and itch. Neuron 85:1289–1304. 10.1016/j.neuron.2015.02.028 25789756 PMC4372258

[B22] François A, Schüetter N, Laffray S, Sanguesa J, Pizzoccaro A, Dubel S, Mantilleri A, Nargeot J, Noël J, Wood JN, Moqrich A, Pongs O, Bourinet E (2015) The low-threshold calcium channel Cav3.2 determines low-threshold mechanoreceptor function. Cell Rep 10:370–380. 10.1016/j.celrep.2014.12.042 25600872

[B23] Fukuoka T, Tokunaga A, Tachibana T, Dai Y, Yamanaka H, Noguchi K (2002) VR1, but not P2X(3), increases in the spared L4 DRG in rats with L5 spinal nerve ligation. Pain 99:111–120. 10.1016/S0304-3959(02)00067-2 12237189

[B24] Gao YJ, Ren WH, Zhang YQ, Zhao ZQ (2004) Contributions of the anterior cingulate cortex and amygdala to pain- and fear-conditioned place avoidance in rats. Pain 110:343–353. 10.1016/j.pain.2004.04.030 15275785

[B25] Honsek SD, Seal RP, Sandkühler J (2015) Presynaptic inhibition of optogenetically identified VGluT3+ sensory fibres by opioids and baclofen. Pain 156:243–251. 10.1097/01.j.pain.0000460304.63948.40 25599445 PMC4299913

[B26] Hunt SP, Mantyh PW (2001) The molecular dynamics of pain control. Nat Rev Neurosci 2:83–91. 10.1038/35053509 11252998

[B27] Ikeda R, Takahashi Y, Inoue K, Kato F (2007) NMDA receptor-independent synaptic plasticity in the central amygdala in the rat model of neuropathic pain. Pain 127:161–172. 10.1016/j.pain.2006.09.003 17055162

[B28] Iyer SM, Montgomery KL, Towne C, Lee SY, Ramakrishnan C, Deisseroth K, Delp SL (2014) Virally mediated optogenetic excitation and inhibition of pain in freely moving nontransgenic mice. Nat Biotechnol 32:274–278. 10.1038/nbt.2834 24531797 PMC3988230

[B29] Ji RR, Baba H, Brenner GJ, Woolf CJ (1999) Nociceptive-specific activation of ERK in spinal neurons contributes to pain hypersensitivity. Nat Neurosci 2:1114–1119. 10.1038/16040 10570489

[B30] Ji ZG, Wang H (2016) ChR2 transgenic animals in peripheral sensory system: sensing light as various sensations. Life Sci 150:95–102. 10.1016/j.lfs.2016.02.057 26903290

[B31] Ji ZG, Ito S, Honjoh T, Ohta H, Ishizuka T, Fukazawa Y, Yawo H (2012) Light-evoked somatosensory perception of transgenic rats that express channelrhodopsin-2 in dorsal root ganglion cells. PLoS One 7:e32699. 10.1371/journal.pone.0032699 22412908 PMC3295764

[B32] Kato G, Furue H, Katafuchi T, Yasaka T, Iwamoto Y, Yoshimura M (2004) Electrophysiological mapping of the nociceptive inputs to the substantia gelatinosa in rat horizontal spinal cord slices. J Physiol 560:303–315. 10.1113/jphysiol.2004.068700 15297573 PMC1665212

[B33] Keller AF, Beggs S, Salter MW, De Koninck Y (2007) Transformation of the output of spinal lamina I neurons after nerve injury and microglia stimulation underlying neuropathic pain. Mol Pain 3:27. 10.1186/1744-8069-3-27 17900333 PMC2093929

[B34] Kim SH, Chung JM (1992) An experimental model for peripheral neuropathy produced by segmental spinal nerve ligation in the rat. Pain 50:355–363. 1333581 10.1016/0304-3959(92)90041-9

[B35] Le Bars D, Dickenson AH, Besson JM (1979) Diffuse noxious inhibitory controls (DNIC). II. Lack of effect on non-convergent neurones, supraspinal involvement and theoretical implications. Pain 6:305–327. 460936 10.1016/0304-3959(79)90050-2

[B36] Le Bars D, Gozariu M, Cadden SW (2001) Animal models of nociception. Pharmacol Rev 53:597–652. 11734620

[B37] Li L, Rutlin M, Abraira VE, Cassidy C, Kus L, Gong S, Jankowski MP, Luo W, Heintz N, Koerber HR, Woodbury CJ, Ginty DD (2011) The functional organization of cutaneous low-threshold mechanosensory neurons. Cell 147:1615–1627. 10.1016/j.cell.2011.11.027 22196735 PMC3262167

[B38] Liljencrantz J, Olausson H (2014) Tactile C fibers and their contributions to pleasant sensations and to tactile allodynia. Front Behav Neurosci 8:37. 10.3389/fnbeh.2014.00037 24639633 PMC3944476

[B39] Lolignier S, Eijkelkamp N, Wood JN (2015) Mechanical allodynia. Pflugers Arch 467:133–139. 10.1007/s00424-014-1532-0 24846747 PMC4281368

[B40] Miraucourt LS, Moisset X, Dallel R, Voisin DL (2009) Glycine inhibitory dysfunction induces a selectively dynamic, morphine-resistant, and neurokinin 1 receptor- independent mechanical allodynia. J Neurosci 29:2519–2527. 10.1523/JNEUROSCI.3923-08.2009 19244526 PMC6666240

[B41] Mishra SK, Tisel SM, Orestes P, Bhangoo SK, Hoon MA (2011) TRPV1-lineage neurons are required for thermal sensation. EMBO J 30:582–593. 10.1038/emboj.2010.325 21139565 PMC3034006

[B42] Montgomery KL, Iyer SM, Christensen AJ, Deisseroth K, Delp SL (2016) Beyond the brain: optogenetic control in the spinal cord and peripheral nervous system. Sci Transl Med 8:337rv335 10.1126/scitranslmed.aad7577 27147590

[B43] Nakatsuka T, Park JS, Kumamoto E, Tamaki T, Yoshimura M (1999) Plastic changes in sensory inputs to rat substantia gelatinosa neurons following peripheral inflammation. Pain 82:39–47. 10422658 10.1016/S0304-3959(99)00037-8

[B44] Nakatsuka T, Ataka T, Kumamoto E, Tamaki T, Yoshimura M (2000) Alteration in synaptic inputs through C-afferent fibers to substantia gelatinosa neurons of the rat spinal dorsal horn during postnatal development. Neuroscience 99:549–556. 11029546 10.1016/s0306-4522(00)00224-4

[B45] Ochoa JL, Yarnitsky D (1993) Mechanical hyperalgesias in neuropathic pain patients: dynamic and static subtypes. Ann Neurol 33:465–472. 10.1002/ana.410330509 8388678

[B46] Peirs C, Seal RP (2016) Neural circuits for pain: recent advances and current views. Science 354:578–584. 10.1126/science.aaf8933 27811268 PMC11327866

[B47] Petitjean H, Pawlowski SA, Fraine SL, Sharif B, Hamad D, Fatima T, Berg J, Brown CM, Jan LY, Ribeiro-da-Silva A, Braz JM, Basbaum AI, Sharif-Naeini R (2015) Dorsal horn parvalbumin neurons are gate-keepers of touch-evoked pain after nerve injury. Cell Rep 13:1246–1257. 10.1016/j.celrep.2015.09.080 26527000 PMC6038918

[B48] Prescott SA, De Koninck Y (2002) Four cell types with distinctive membrane properties and morphologies in lamina I of the spinal dorsal horn of the adult rat. J Physiol 539:817–836. 10.1113/jphysiol.2001.013437 11897852 PMC2290183

[B49] Prescott SA, Ma Q, De Koninck Y (2014) Normal and abnormal coding of somatosensory stimuli causing pain. Nat Neurosci 17:183–191. 10.1038/nn.3629 24473266 PMC4079041

[B50] Ruscheweyh R, Sandkühler J (2002) Lamina-specific membrane and discharge properties of rat spinal dorsal horn neurones in vitro. J Physiol 541:231–244. 12015432 10.1113/jphysiol.2002.017756 PMC2290304

[B51] Ruscheweyh R, Ikeda H, Heinke B, Sandkühler J (2004) Distinctive membrane and discharge properties of rat spinal lamina I projection neurones in vitro. J Physiol 555:527–543. 10.1113/jphysiol.2003.054049 14694142 PMC1664848

[B52] Sandkühler J (2009) Models and mechanisms of hyperalgesia and allodynia. Physiol Rev 89:707–758. 10.1152/physrev.00025.2008 19342617

[B53] Shen J, Fox LE, Cheng J (2012) Differential effects of peripheral versus central coadministration of QX-314 and capsaicin on neuropathic pain in rats. Anesthesiology 117:365–380. 10.1097/ALN.0b013e318260de41 22739765 PMC3421838

[B54] Sugimura YK, Takahashi Y, Watabe AM, Kato F (2016) Synaptic and network consequences of monosynaptic nociceptive inputs of parabrachial nucleus origin in the central amygdala. J Neurophysiol 115:2721–2739. 10.1152/jn.00946.2015 26888105 PMC4922599

[B55] Tarpley JW, Kohler MG, Martin WJ (2004) The behavioral and neuroanatomical effects of IB4-saporin treatment in rat models of nociceptive and neuropathic pain. Brain Res 1029:65–76. 10.1016/j.brainres.2004.09.027 15533317

[B56] Todd AJ (2010) Neuronal circuitry for pain processing in the dorsal horn. Nat Rev Neurosci 11:823–836. 10.1038/nrn2947 21068766 PMC3277941

[B57] Tomita H, Sugano E, Fukazawa Y, Isago H, Sugiyama Y, Hiroi T, Ishizuka T, Mushiake H, Kato M, Hirabayashi M, Shigemoto R, Yawo H, Tamai M (2009) Visual properties of transgenic rats harboring the channelrhodopsin-2 gene regulated by the thy-1.2 promoter. PLoS One 4:e7679. 10.1371/journal.pone.0007679 19893752 PMC2772120

[B58] Tsuda M, Shigemoto-Mogami Y, Koizumi S, Mizokoshi A, Kohsaka S, Salter MW, Inoue K (2003) P2X4 receptors induced in spinal microglia gate tactile allodynia after nerve injury. Nature 424:778–783. 10.1038/nature01786 12917686

[B59] Tsuda M, Masuda T, Kitano J, Shimoyama H, Tozaki-Saitoh H, Inoue K (2009) IFN-gamma receptor signaling mediates spinal microglia activation driving neuropathic pain. Proc Natl Acad Sci USA 106:8032–8037. 10.1073/pnas.0810420106 19380717 PMC2683100

[B60] Tsuda M, Kohro Y, Yano T, Tsujikawa T, Kitano J, Tozaki-Saitoh H, Koyanagi S, Ohdo S, Ji RR, Salter MW, Inoue K (2011) JAK-STAT3 pathway regulates spinal astrocyte proliferation and neuropathic pain maintenance in rats. Brain 134:1127–1139. 10.1093/brain/awr025 21371995 PMC4571138

[B61] Usoskin D, Furlan A, Islam S, Abdo H, Lönnerberg P, Lou D, Hjerling-Leffler J, Haeggström J, Kharchenko O, Kharchenko PV, Linnarsson S, Ernfors P (2015) Unbiased classification of sensory neuron types by large-scale single-cell RNA sequencing. Nat Neurosci 18:145–153. 10.1038/nn.3881 25420068

[B62] Xu ZZ, Kim YH, Bang S, Zhang Y, Berta T, Wang F, Oh SB, Ji RR (2015) Inhibition of mechanical allodynia in neuropathic pain by TLR5-mediated A-fiber blockade. Nat Med 21:1326–1331. 10.1038/nm.3978 26479925 PMC4752254

[B63] Yamamoto W, Sugiura A, Nakazato-Imasato E, Kita Y (2008) Characterization of primary sensory neurons mediating static and dynamic allodynia in rat chronic constriction injury model. J Pharm Pharmacol 60:717–722. 10.1211/jpp.60.6.0006 18498707

[B64] Yasaka T, Kato G, Furue H, Rashid MH, Sonohata M, Tamae A, Murata Y, Masuko S, Yoshimura M (2007) Cell-type-specific excitatory and inhibitory circuits involving primary afferents in the substantia gelatinosa of the rat spinal dorsal horn in vitro. J Physiol 581:603–618. 10.1113/jphysiol.2006.123919 17347278 PMC2075204

[B65] Yoshimura M, Nishi S (1993) Blind patch-clamp recordings from substantia gelatinosa neurons in adult rat spinal cord slices: pharmacological properties of synaptic currents. Neuroscience 53:519–526. 8098516 10.1016/0306-4522(93)90216-3

